# Sciatic Neurectomy in the Horse

**Published:** 1899-04

**Authors:** 


### CLINICAL REPORTS.

### VETERINARY HOSPITAL, UNIVERSITY OF PENNSYLVANIA.

Clinic of S. J. J. Harger, V.M.D.,
PROFESSOR OF VETERINARY ANATOMY AND ZOÖTECHNICS,
AND
J. H. McNeall, V.M.D.,
RESIDENT SURGEON.

Sciatic Neurectomy in the Horse. The subject was a gray draught gelding, which had been lame for four months. The lameness resulted from what is ordinarily called a severe "strain" of the near hind fetlock. The parts from the middle of the canon to the lower end of the first phalanx were swollen to about three times their normal volume; there was no anchylosis, but a slight mechanical interference with the mobility of the articulation. The fetlock was blistered, but a day's work made him so lame that he had to be brought to the hospital in an ambulance. After some days of rest, in order to allay the inflammatory symptoms, sciatic neurectomy was decided upon.

The sciatic nerve passes downward on the inside of the tibial region parallel with but a short distance anterior to the tendon of Achilles, the point of election being about one and one-half inches in front of the posterior profile of the leg and about four inches above the point of the hock. Resection was made in the ordinary manner.

The animal, now ready to be discharged, still showed a slight lameness. This fact, however, can be anticipated, for two reasons: there is undoubtedly some mechanical interference with the articular movements, also, the external saphena and anterior tibial nerves supply the lateral and anterior regions of the canon and phalanges; the sensibility thus retained in these parts may contribute to whatever lameness exists. The animal will very likely continue to improve with time, and is in such a condition that he will be able to work. The actual cautery, of course, suggested itself, but the prognosis was very doubtful and it would have required a long time for the animal to recover.

Sciatic neurectomy may suggest itself for the relief of the same condition in the hind foot for which the median operation is practiced in the fore foot.

The question may be asked as to whether any trophic changes follow this operation—a condition which must always be thought of. Upon this point I cannot write from experience, but in this respect the sciatic operation may, perhaps, not be as safe as the median, because in the latter a certain amount of sensibility is retained on the external side of the foot. It is, however, equally as safe as the combined median and ulnar operations sometimes recommended and found to answer the purpose.

